# Epigenetic Regulations of AhR in the Aspect of Immunomodulation

**DOI:** 10.3390/ijms21176404

**Published:** 2020-09-03

**Authors:** Anna Wajda, Joanna Łapczuk-Romańska, Agnieszka Paradowska-Gorycka

**Affiliations:** 1 Department of Molecular Biology, National Institute of Geriatrics, Rheumatology and Rehabilitation, 02-637 Warsaw, Poland; paradowska_aga@interia.pl; 2 Department of Experimental and Clinical Pharmacology, Pomeranian Medical University, 70-111 Szczecin, Poland; joanna.lapczuk@pum.edu.pl

**Keywords:** AhR, epigenetic, immunomodulation, autoimmunity

## Abstract

Environmental factors contribute to autoimmune disease manifestation, and as regarded today, AhR has become an important factor in studies of immunomodulation. Besides immunological aspects, AhR also plays a role in pharmacological, toxicological and many other physiological processes such as adaptive metabolism. In recent years, epigenetic mechanisms have provided new insight into gene regulation and reveal a new contribution to autoimmune disease pathogenesis. DNA methylation, histone modifications, chromatin alterations, microRNA and consequently non-genetic changes in phenotypes connect with environmental factors. Increasing data reveals AhR cross-roads with the most significant in immunology pathways. Although study on epigenetic modulations in autoimmune diseases is still not well understood, therefore future research will help us understand their pathophysiology and help to find new therapeutic strategies. Present literature review sheds the light on the common ground between remodeling chromatin compounds and autoimmune antibodies used in diagnostics. In the proposed review we summarize recent findings that describe epigenetic factors which regulate AhR activity and impact diverse immunological responses and pathological changes.

## 1. Introduction

Heredity is only the sum of all past environment—this famous statement published in 1906 in the article “The Training of the Human Plant” in Century magazine by Luther Burbank [1] has never been so much reliable and evident as in recent years when epigenetics and its underlying mechanisms have changed classical approach to many topics requiring a thorough understanding of all aspects of genetics, such as stem cells, cloning, aging or synthetic biology [2]. Epigenetic mechanisms, defined as the mitotically heritable changes, regulate or affect gene expression without affecting genome sequence [3] by methylation of DNA, modification by non-coding RNA [4], histone modification, and chromatin remodeling [4,5]. More and more studies reveal that in the case of complex autoinflammatory disorders, non-genetic mechanisms lead to pathophysiological responses. In accordance with the recent literature, there is a clear correlation between environmental factors and diseases [6]. In the case of those kinds of pathological conditions also well-established heritability model is problematic and very often it is a subject of controversy [5]. In this respect, more in-depth research providing epigenetic studies could be a great value in searching for potential biomarkers, molecular mechanisms responsible for maintaining balance in autoimmunity, and crucial in clinical aspects such as efficient drug therapy. Therefore, in recent years, there has been an increasing focus on epigenetic changes in autoimmune diseases [7]. Additionally, it becomes clear that environmental pollutants, diet and lifestyle are linked with epigenetic variations, including changes in DNA methylation, histone modifications and microRNA [5,8].

It has been estimated that autoimmune diseases (ADs) represent a family of at least 80 illnesses that share common pathogenesis [9]. It is not possible within the scope of this article to provide a comprehensive overview of all ADs, therefore we referred to the selected examples which are well described in the aspect of aryl hydrocarbon receptor (AhR) and epigenetic regulation (Table 1).

Considering the particular role of AhR in immune responses, increasing data reveals its cross-roads on the other significant in immunology pathways such as NF-κB [10], TGFβ/SMAD3 [11,12,13] and TLRs [14]. For example, the AHR seems to regulate innate inflammatory signaling not only through binding to its cognate response element in association with ARNT but also through direct binding to RELA (also known as p65) and RELB, which are members of the NF-κB family of transcription factors [15,16]. AhR interacts with ERα (estrogen receptor α, present in immune cells) but also directly binds estradiol [17,18,19]. Additionally, cross-talk between ER and NF-κB has been demonstrated in many studies [20]. Recently, Shinde et al. [21] revealed that AhR is activated by TLR9 and DNA from apoptotic cells in systemic lupus erythematosus patients (SLE) [21]. This assumption that TLR ligands and cytokines are potent agonists in various cell types has been pointed even a few years back by other authors [22,23]. 

### 1.1. AhR Regulation in Immune System

**Figure 1:**
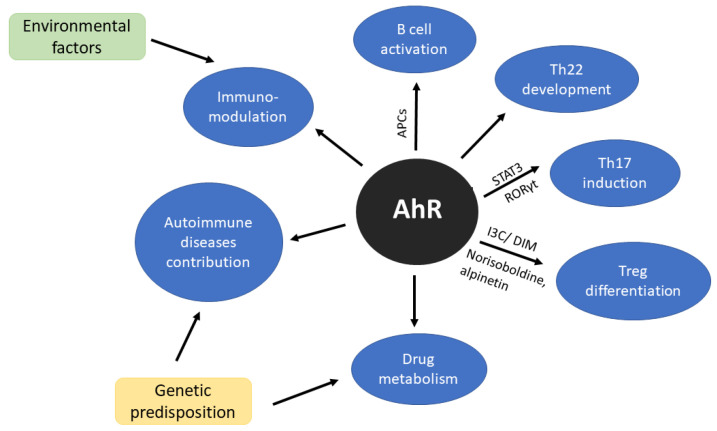
Scheme of aryl hydrocarbon receptor (AhR) significance in immune system. AhR as a sensor of environmental factors affects immunomodulation. Additionally, genetic compound may trigger the mechanisms of autoimmune diseases development and treatment response.

Aryl Hydrocarbon Receptor (AhR) is one of the transcription factors that has been extensively studied in the context of cellular responses to environmental toxins and also plays an important physiological role in T cell immune responses and T cell differentiation (Figure 1) [68]. Together with signal transducer and activator of transcription factor 3 (STAT3) and retinoid-related orphan receptor gamma (RORγt) is crucial in Th22 induction [69]. AhR is a modulator of B cell activity [70,71], furthermore, cells presenting antigen (CPAs) also express elevated level of AhR after stimulation [30,72]. For many years Th22 was considered as a subtype of Th17 but Miyazaki et al. [73] study do not support this hypothesis. Moreover, their research suggests that Th22 may be directly involved in joint destruction [73]. For more detailed information on AhR regulation in immune response, we would recommend recent reviews written by Gutiereez-Vazquez and Qiotana [74] or Trikha and Lee [75]. 

### 1.2. AhR Ligands

AhR, a basic helix-loop-helix/Per-Arnt-Sim (bHLH/PAS) transcription factor [76,77], is best known for mediating of the biotransformation and cancerogenic/teratogenic effects of environmental toxins such as 2,3,7,8-tetrachloro- dibenzo-p-dioxin (TCDD) [77,78] or many polycyclic aromatic hydrocarbons (PAHs) such as benzo-A-pyrene, dimethylbenzanthracene [78]. AhR regulates phase I and II drug-metabolizing enzymes [79] and drug transporters [80]. In accordance with that environmental factors undoubtedly contribute to autoimmune disease manifestation [6], and as regarded today, AhR has become an essential factor in studies of immunomodulation [81]. Moreover, epidemiologic studies suggested that rates of autoimmune diseases are increasing, particularly in industrialized countries [9] and most prevalent in women [82]. Interestingly, xenoestrogens are endocrine disruptors and many of them have an impact on AhR activation [83]. 

In the absence of a ligand, AhR is localized in the cytoplasm of the cell and associated with a dimer of Hsp90 and other proteins such as p23, immunophilin-like AhR interacting protein AIP (also known as XAP2 or Ara9) or cytosolic proteins such as kinome chaperone Cdc37 and the non-receptor tyrosine kinase Src [76,77,84]. Ligand binding leads to the conformational changes and as a consequence AhR dissociates from Hsp90, translocates to the nucleus and heterodimerizes with ARNT (another bHLH-PAS protein which constitutively occurs in the nucleus). The AhR-ARNT complex binds to promoters AhR gene responsive elements referred (AhRE), dioxin response elements (DRE), or xenobiotic response elements (XRE) and initiates or regulates target gene transcription [6,77]. Moreover, many studies revealed an alternative pathway of activated AhR signaling and cross-talk or competition for transcription cofactors such as NF- NF-kB, ER or NRF2 [85,86].

AhR activity may be regulated via a few different mechanisms. One of them is degradation by the proteasome. Binding Ahr with ligand induces ubiquitination and subsequent degradation. Another is the auto-regulatory feedback loop. AhR activation induces the expression of negative regulators, which prevent excessive AhR activation. For example, a repressor of AhR–AhRR through competition with AhR-ARNT complex downregulates the expression of AhR dependent genes [77]; or few years ago discovered, more general regulator TiPARP (TCDD-induced poly-ADP-ribose polymerase, ARTD) [87]. Moreover, indirect regulation by the availability of AhR ligands may also suppress activity, for example, by the induction of ligand metabolizing enzymes such as CYP1 and consequently by the prevention of prolonged AhR activation [77]. In recent years, epigenetic mechanisms, which are presented in this paper, have provided new insight into AhR regulation. 

It should be mentioned that AhR activation and its target gene networks may be caused not only by agonist or antagonist but also by factors that do not fit well into the AhR ligand-binding domain (LBD) and by numerous stress factors like oxidative stress, shear stress that do not bind at all with the LBD [88]. Nevertheless, as many studies showed, besides different ligand-dependent AhR responses, also the tissue or even cell type determines the level of expression and signaling pathway [6]. However, up to date, a list of diverse AhR ligands is impressive and still growing. Additionally, advances in the microbiota research suggest that microflora and their metabolic products can activate AhR [77] and shape immunity and tolerance [88]. Recent discoveries also complement epigenetic regulation, for example, butyrate (short-chain fatty acid derived from bacterial fermentation of dietary fibers) stimulates AhR-dependent genes probably by decreasing expression of histone deacetylase (HDAC) in the human intestinal cell [89].

In general, according to chemical structure, AhR ligands may be categorized into two major classes. The first one, which was mentioned above, contains aromatic hydrocarbons, which are derived mainly from environmental toxins [90] or also through the medicine or diet [79]. 

Another class of AhR ligands is composed of tryptophan-based molecules that can be derived from the diet or endogenously produced by the host organism [90] such as 6-formylindolo[3,2-b]carbazole (FICZ), indolo-[3,2-b]-carbazole (ICZ, converted from indole-3-carbinal which is found at especially high levels in cruciferous vegetables [91]) or kynurenine [77], cinnabarinic acid [92], galic acid (3,4,5-trihydroxybenzoic acid–metabolite of shikimate pathway) [93]. Generally, FICZ is an ultraviolet photoproduct of L-tryptophan, but it can also be generated during other metabolic pathways such as tryptamine deamination or tryptophan oxidation by intracellular oxidants [78,91]. As compared to dioxins, FICZ reveals different, rapid kinetic patterns of AhR responses, which lead to low intracellular steady-state levels of FICZ. Its high specificity and observed catalytic efficiencies support the argument that this molecule is an endogenous AhR ligand of high importance [6]. Well-established immunoregulatory IDO activity and tryptophan metabolites participation in the regulation of many cell types, including T cells, DC, monocytes, macrophages, and so forth, has been reviewed by Ngyuenet et al. [22]. In large scale pharmaceuticals screening published by Hu et al. [94] leflunomide appeared to be the most potent AhR agonist. Additionally, nimodipine (a calcium-channel blocker indicated for subarachnoid hemorrhage) and flutamide (an androgen receptor antagonist indicated for prostate cancer) for the first time were reported as compounds which competitively bind to the AhR in vitro [94]. Other studies also showed that some benzimidazole derivatives, antiallergenic drugs, are AhR receptor agonists in vivo and in vitro [95,96]. Recently, more and more evidence shows that norisoboldine (an alkaloid from Radix linderae) is AhR ligand and have an impact on Treg differentiation. As a herb-drug and AhR ligand, norisoboldine attenuates rheumatoid arthritis (RA) and colitis [97,98]. 

Among endogenous AhR ligands such as bilirubin and biliverdin, prostaglandin G2, indirubin and lipoxin A4 have been identified. In the case of endogenous ligands, rapid metabolism is very often observed, also as a negative feedback loop mechanism. For example, anti-inflammatory lipoxin A4 is degraded by Cytochrome P450, family 1, subfamily A, polypeptide 1 (CYP1A1) and bilirubin via UDP glucuronosyltransferase family 1 member A1 (UGT1A1), whose expression is induced by AhR [99]. As it was mentioned before, the physiological effect may be produced even by ligands with relatively weak affinity for binding the AhR, such as genistein (flavonoid present in soy), and quercetin (flavonol in apples, tea, onions) because of their wide occurrence in the diet [78]. 

## 2. Crosstalk between AhR and DNA Methylation 

DNA methylation was the first described covalent DNA modification. In this process methyl group onto the 5’ carbon position of cytosine in CpG islands is added and in consequence access to DNA for transcription factors and RNA polymerases is reduced and transcription is repressed [100]. DNA methylation plays a role in promoting the proinflammatory immune cells, which might depend on a large extent on environmental factors (UV exposure, viral infection) but also individual factors (genetic predisposition, age, diet, etc.) and medications (DMARDs-disease-modifying anti-rheumatic drugs) [101]. Decreased global DNA methylation has been observed in immune cells from SLE patients [102], in PBMC and CD4+ T cells of psoriasis patients [42,103] whereas in Sjögren Syndrome, in epithelial cell of the salivary gland and minor salivary glands hypomethylation and reduced DNA methyltransferase (DNMT) level was observed [47,104]. 

Research conducted on Atlantic Killifish (*Fundulus heteroclitus*) that adapted to survive in PCB-contaminated water, revealed an evolved genetic mechanism where methylation status of promoter regions may control the tissue-specific expression of AhR genes. PCB-resistant and PCB-sensitive populations of killifish respectively, exhibit different patterns of DNA methylation in the promoters of AhR genes [105]. It also appears that early life activation of AhR alters the responsive capacity of CD8+ T cells and consequently influences the interaction between DNA methylation and gene expression during infection [106]. 

In 2006, Mulero-Navarro et al. described for the first time a case of methylated AhR promoter in human acute lymphoblastic leukemia [107]. Methylation of AhR CpG sequences is responsible for AhR activation, however, this regulation seems to be cell-type specific. 

Andrade et al. [108] conducted a study showing the connection between methylation of AhR and its clinical implementation. It has been revealed that contrary to normal cells, in acute lymphoblastic leukemia (ALL) AhR promoter gene is methylated and consequently deactivated. Although the majority of patients with ALL respond well to current therapies about 20% of affected children relapse or have a poor prognosis because of the development of drug resistance. The authors showed that DNA demethylation by zebularine (1-[b-D-ribofuranosyl]-1,2-dihydropyrimidin- 2-one; ZB) in primarily resistant cells can sensitize cells to methotrexate (MTX) and re-express the AhR gene. ZB also caused apoptosis and re-expressed the silenced methylated AhR. Additionally, ZB combined with MTX synergistically affected leukemia cell lines by decreasing their growth and clonogenic capacity. Demethylation and activation of AhR may contribute to restoring normal cell phenotype and prevent ALL development [108]. In contrast to studies on leukemia cells, mechanically different AhR silencing has been noted in MCF-7 or -10A breast cancer cells. In this case, AhR silencing probably is not connected with DNA methylation but histone modification, which is another element of epigenetic modulation [109]. Depending on AhR ligands, process of DNA methylation might be modulated as it has been observed for Treg/Th17 differentiation process. In CD4+ T cells from lupus patients, low activity of DNMT1 has been observed after activation of AhR by UVB. AhR suppresses SIRT1 which has a negative effect on DNTM1 activity [110]. Liu et at. [111] revealed that activation of AhR by phenanthrene leads to conversion of primary human Treg cells to Th2 cells, whereas TCDD and FICZ to Th17 cells. Moreover, inhibition of AhR results with significant FOXP3 expression, decrease of CpG sites methylation in its locus and reduce DNA (cytosine-5)-methyltransferase (DNMT)1 and DNMT3b transcripts. Interestingly, additional treatment of Treg cells with transforming growth factor β (TGF-β), partially reverses methylation of FOXP3 and maintains Treg phenotype [111]. TCDD-activated AhR displays to partially demethylate CpG islands of FOXP3 and to hypermethylate IL-17 promoters in colitis [51]. Liu et al. [111] study is significant in understanding the pathophysiology of atopic diseases such as atopic dermatitis, asthma, and hay fever and their association with Th2 and environmental pollution. Although the literature is not consistent on AhR ligand specificity and T cell differentiation [51], the relationship between AhR activity and methylation seems to be in line. Activation of AhR decreases the level of DNMT expression, which has been proved in many studies [112,113]. This hypothesis correlates with demethylation mechanism. Research on hepatocytes indicated that AhR is important in the bare excision repair (BER) pathway where methylated cytosine is replaced by non-methylated in CYP1a1 promoter and increase mRNA level of CYP1a1 [114]. 

AhR also plays a role in B cell differentiation. Recently, it has been proved that in induce arthritis in mice model, AhR promoter is methylated in B cell and its demethylation by 5’-azacytidine decrease IgG antibody production. Another consequence of reactivation of AhR is the suppression of *Aicda* (single-stranded DNA-specific cytidine deaminase) expression, crucial for efficient antibody response [115]. Overproduced IgG1 antibodies form immune complexes on the articular cartilage surface and contribute to joint destruction in RA pathogenesis [116]. It has been shown that AhR expression is also downregulated in PBMC and B cells in treatment naïve RA patients. However, hypermethylation was not observed like in mice model of RA in promoter AhR but in the distant intergenic region, precisely 155 kb upstream of AhR transcription start site. Nevertheless, in both experiments, AhR was silenced. Therefore, the authors postulated that treatment with DNA methyltransferase inhibitor may have a therapeutic potential in autoimmune arthritis [115]. However, on the other hand, 5-azacytidine may induce lupus-like disease in rodents [117,118]. 

## 3. Post-Translation Histone Modification (Methylation, Acetylation of Residues in the N-Terminal Tails of Histones)

Chromatin remodeling and histone modification are interrelated processes. Regulation of gene expression is strongly associated with the state of chromatin—1) euchromatin-relaxed with active genes and 2) packed, transcriptionally inactive heterochromatin [119]. The basic structural unit of the chromatin is nucleosome consisted of DNA and histones. Histone modification constitutes of many diverse classes and complex regulations and the histone tails are the targets of changes [100]. Several modifications of histone have been identified as regulators of gene expression—acetylation, methylation, phosphorylation, ubiquitination, ADP-ribosylation, and sumoylation. An additional aspect is that histones variants may contribute to the chromatin changes [120] and typically, each variant has dedicated chaperones [121]. Antibodies against the centromere-specific histone H3-variant, so-called anti-CENP-A, are generated in patients suffering from for example, systemic sclerosis (SSc) but not in healthy people and cancer patients [122]. 

In the present review, we focused particularly on methylation and acetylation as they have been mostly studied in the aspect of AhR and immune regulations. Histone methylation affects lysine (K) and arginine (R) residues. Lysine can be methylated with one to three methyl groups, while arginine can be methylated with only one methyl group. Histone methylation correlates with gene activation and repression. An active form of chromatin consists with for example, H3K4me; H3K36me, H3K79me while H3K9me2 and H3K27me3 occur in condensed chromatin and silence the activity of genes. Histone methylation is conducted by the histone methyltransferases HKMT and PRMT whereas demethylation by histone demethylases (HDM) [123,124,125]. During histone acetylation acetyl group is attached to the lysine and the reaction is catalyzed by the histone acetyltransferases HATs. Some of the proteins such as p55 or p300/CBP, which have been known of their other biological function before, appeared to have HATs activities. HATs active in the nucleus belongs to HAT A type, whereas those localize in the cytoplasm (e.g., HAT1) belongs to HAT B type [126]. This process leads to chromatin decondensation and consequently activates transcription. The reverse process associated with chromatin condensation and transcription inhibition is deacetylation of histones with histone deacetyltransferases HDACs. Noteworthy is that the modification of histones is dynamic, therefore gene transcription might be epigenetically regulated [127].

**Figure 2:**
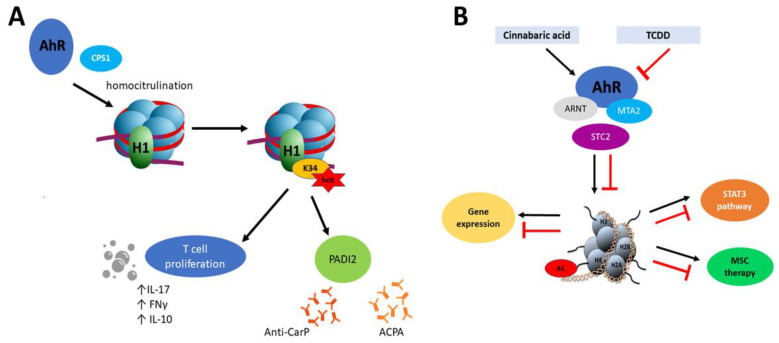
(**A**) Mechanism of homocitrulination and anti-CarP and anti-citrullinated protein (ACPA) antibody production. AhR recruits CPS1, which is responsible for homocitrulination of H1K34. H1K34*hct* enhances PADI2 expression, affects proliferation of T cells and production of IL-10 and the pro-inflammatory cytokines IFN-γ and IL-17. PADI2 plays a role in anti-CarP and ACPA antibody production. (**B**) Histone acetylation mediated by AhR. Cinnabaric acid (but not 2,3,7,8-tetrachloro- dibenzo-p-dioxin (TCDD)) activates AhR which recruits MTA2. Complex AhR/ARNT -MTA2 leads to transcriptional activation of STC2. MTA2 takes part in acetylation of histone 4. STC2 reveals anti-inflammatory properties which have been implicated into MSC therapy. STC2 also activates STAT3 signaling.

However, epigenetic mechanisms of AhR and the role of this transcription factor in epigenetic modifications are not fully explored. For example, Joshi et al. [127] showed that AhR recruits carbamoyl phosphate synthase 1 (CPS1) which is responsible for homocitrullination (carbamylation) of histone H1 on lysine 34 (H1K34). Moreover, H1K34hcit is implicated in the enhanced expression of the peptidyl arginine deiminase 2 gene (PADI2) [127]. Carbamylation is a post-translational modification of proteins which similarly to citrullination seems to be implicated in the pathogenesis of rheumatoid arthritis [128] but also with Sjögren Syndrome [129]. In the inflammation process, carbamylated lysine affects proliferation of CD4+ T cells, production of IL-10 and the pro-inflammatory cytokines IFN-γ and IL-17 in combination with a response to antibodies (anti-CarP) [130]. The process described by Joshi et al. [127] combines PADI 2- expression in an AhR-dependent manner. It is also noteworthy that PADs proteins, including PADI2, facilitate chromatin decondensation during NET formation (neutrophil cellular trap) which along with DNA and citrullinated histones are one of the significant factors during the developing autoimmunity. Similarly to carbamylation, histone citrullination results in inflammation process by activation of immune T cells response and induction of specific antibodies (ACPA) [131]. This cascade has been particularly described in RA pathogenesis, where NET activates synovial fibroblast with proinflammatory response and production of citrullinated autoantigens [132] (Figure 2A). 

Global hypermethylation (except H3K4 methylation) and also novel methylation marks have been observed in a lupus-prone mouse study [133]. Hypermethylation H3K4me3 along with methylation CpG sites in CD4+ and CD8+ was marked and their association with several T cell signaling genes also in Graves’ disease [67]. Many autoimmune diseases are triggered with IFNα [134,135], according to this, Stefan et al. [136] checked the IFNα effect on epigenetic changes in thyroid cells. It has appeared that IFNα can lead to raised methylation level of H3K4me what is associated with the lower TSHR expression. As a consequence, autoantigen expression in the thymus decreased, which lead to the escape of self-reactive T cells [136]. Epigenetic analysis in case of type 1 diabetes revealed that high level of methylation H3K9me in CTLA4 (T1D susceptibility gene) correlates with T cell activation [63]. Study on SSc revealed a lower level of methylation in H3K27me3 in CD4+ cells [137]. 

P300, one of the major HAT proteins, has an impact on STAT3 expression and consequently IL10 production and B cell activity, and production of specific autoantibodies in SLE [28]. In T cell of SLE patients, increased level of transcription factor CREMα (cAMP response element modulator) was observed. According to the study by Tenbrock et al. [138], CREMα interacts with HDAC1 and terminates IL-2 production. A similar mechanism has been observed also in monocytes, macrophages and B cells [138]. A recent clinical trial with low-dose IL-2 treatment resulted in a higher rate of complete remission in patients with lupus nephritis. Additionally, low-dose IL-2 therapy promotes regulatory T cells and may also sustain cellular immunity with enhanced natural killer cells [139].

In the histone acetylation process, the electrostatic interaction between histones and DNA is diminished. Consequently, the chromatin structure is decondensed which provides access to the transcription initiation [140]. It appears that histone acetylation is crucial for the activation of AhR promoter. Inhibitors of histone deacetylase 1 (HDAC1), such as n-butyrate and trichostatin A (TSA), may suppress AhR expression [35]. On the other hand, AhR may have an impact on local histone hyperacetylation and methylation by interaction with coactivators or by displacing histone deacetylase (HDAC) complexes [141]. 

It has been established that in human T cells, HAT and HADC enzymes co-occupy active and aligned genomic loci to maintain proper transcription regulation and uncontrolled binding of RNA polymerase II [142]. 

Hyperacetylation has been reported in RA patients, which is related to HATs and HDACs disbalance [33]. High acetylation H3ac results with high expression of IL-6 in synovial fibroblast in RA [32]. Similarly, in SSc fibroblast hypoacetylation of H3 and H4 was reported [40]. In the case of Graves’ disease high acetylation of H3K27ac in CD4+ and CD8+ in PBMC [66,67] was observed but low H4ac in PBMC [66]. A concept of a ‘‘transcription cycle’’ model in the epigenetic control, observed in case of CYP1A1 transcriptional activity through histone modifications, has been proposed by Tian [10]. The author focused on AhR–NF-kB interaction and described to the positive and negative regulation of transcription by these two factors. Briefly, PRMT1 is a co-activator of AhR and PRMT1 takes part in arginine methylation of H4R3. Methylation is crucial for transcription but also for acetylation of H4 which blocked methylation of H43R. To activate methylation, deacetylation process of H4 is necessary with the HDAC activity. Interestingly, this mechanism occurs after TCCD treatment whereas exposition to TNFalpha (NF-kB regulator) reveals the opposite cascade of reactions [10]. Likewise, other histone acetylase coactivators such as p300, SRC1 and -2, and p300/CBP-associated factor are triggered after TCDD induction [143,144]]. 

Based on the earlier study, it seems that HDAC inhibitors may be used in successful autoimmune diseases therapy. It has been shown that the HDAC inhibitors (e.g., trichostatin A) cause changes in T cells and reduces the activity of SLE [145]. Also studies in an animal model of RA have demonstrated anti-inflammatory properties of HDACi in synovial fibroblasts. HDAC2/3-selective inhibitor reduces IL-6 production in RA patients. Interestingly, conventional treatment with TNFalpha inhibitor did not affect HDAC activity in PBMC from RA patients [146]. Other examples of efficient treatment with HDAC inhibitor are largazole in RA [147] or givinostat in juvenile idiopathic arthritis [148]. In the case of SSc this kind of therapy revealed decrease of collagen production [149,150]. 

Depending on the AhR ligand, a different response might be observed. Joshi et al. [92] revealed that Stc2 (stanniocalcin 2), a target AhR gene, is expressed after activation of AhR by cinnabarinic acid (endogenous ligand, a metabolite of tryptophan in kynurenine pathway) but not after TCDD interaction (Figure 2B). Additionally, in this mechanism, the new complex between AhR-ARNT with MTA2 (metastasis tumor-associated protein 2) has been described. MTA2 recruitment is necessary for histone H4 acetylation at positions K5, -8, -12, and -16. In the aspect of epigenetic regulation, this is interesting because MTA2 is a chromatin-modifying protein and component of the nucleosome remodeling and deacetylation (NuRD) complex. MTA2 may repress and as well activate gene expression [92]. Stanniocalcins (STC) belong to anti-inflammatory proteins and it has been proved that STC2 is significant in MSC (mesenchymal stromal cell) therapy in allergic contact dermatitis [151]. Knockdown of STC2 in MSC was associated with reducing TNF-α- and IFN-γ-producing CD8+ T cells [151], lower production of anti-inflammatory IL10 by MSC, and impaired macrophage polarization from monocytes THP1 cells [152]. mRNA level of STC2 was significantly higher in the intestinal mucosa of CD patients compared to controls [153]. Additionally, STC2 activates also the STAT3 signaling pathway [154]. 

## 4. Chromatin Remodeling

Chromatin remodeling is usually initiated by posttranslational modification of the amino acids that make up the histone proteins, but also through the methylation of neighboring DNA. Additionally, proper heterochromatin formation in certain regions of the mammalian genome requires Dicer- an enzyme involved in microRNA maturation [155]. One of the most widely used methods is chromatin immunoprecipitation (ChIP) assay. Chromatin is sheared and immunoprecipitated with a specific antibody directed against a component of the chromatin complex. Other methods that enable analysis of various histones modification within one nucleosome is mass spectrometry, however, due to methodology, it seems that proteomic analysis will be more functional [140].

Chromatin remodeling is a process in which a cascade of protein complexes, transcription factors, coactivators participate and together with RNA polymerase II (Pol II) enables transcription initiation. So-called “bridge model” connects Mediator, large multisubunit polypeptides with the transcription factors and Pol II. Mediator complex might be a master in epigenetic regulation, but also a “coordinator” in non-coding RNA activation or super-enhancer formation [156]. It has been shown that one of the Mediator subunits, Med220 enhances AhR/ARNT-dependent transcription of CYP1A1 in different mechanistically way. Contrary to the CYP1A1 expression with the interaction of AhR and p300, Med220 associate with the enhancer, not with the promoter of CYP1A1. Therefore, these two different regulations of CYP1A1 expression are characterized by different kinetics. Variety of mediator complex in tissues helps to explain the alteration in response to for example, chemical toxicity and different susceptibility of cells/tissues [157]. Recently, a more complex association involving the Mediator subunit Med1 (MED1) and AhR has been proven by Chowdhary et al. [158] who described a mechanism of chromatin remodeling with an additional microRNA involvement. Although the study is specific for hepatocytes and is associated with acetaminophen toxicity, it sheds the light on very complex machinery of epigenetic regulations and strict dividing them into the individual process is sometimes impossible. Chowdhary et al. [158] showed that miR-122 protects hepatocytes from toxicity caused by acetaminophen. Additionally, it appears that miR-122 regulates CYP1A2 expression through AhR, MED1 and another transcription factor CTFC. Both CTFC and MED1 are target for miR-122 and low levels of this microRNA and high activity of AhR-mediated CYP1A2 are observed in the liver failure. AhR or Med1 depletion is associated with downregulation at mRNA and protein levels of Cyp1a2 in mouse liver. However, the regulation of AhR and MED1 expression by mir-122 occurs by distinct mechanisms. Contrary to Med1, mouse AhR does not have a binding site for miR-122 and is not directly regulated by this microRNA [158]. 

Another complex of proteins SWI/SNF (also known as BRG1/BRM-associated factor (BAF) complexes) with other histone-modifying enzymes may independently or cooperatively mediate remodeling of targeted chromatin structures [159]. Nowadays, we know that chromatin remodeling complexes such as SWI/SNF and NuRD (nucleosome remodeling and deacetylase) are associated with Ikaros. First reports on Ikaros–a zinc-finger DNA binding protein as an enhancer and promoter elements critical for the expression of lymphoid-specific genes has been written in 90’ [160,161]. Ikaros plays a critical role in hematopoiesis and B-cell differentiation, its deficiency caused immunodeficiency type 13 [162]. Moreover, Ikaros is required to limit repressive chromatin modifications at Ahr, Runx1, Rorc, Il17a, and Il22 loci, thus maintaining the potential for expression of the Th17 gene program. It binds directly to the AhR and Runx1 promoter which consequently control expression of Rorc and Il17a, therefore it can be concluded that Th17 development is controlled by Ikaros in direct chromatin modulation in AhR and Runx1 genes [163].

AhR activity is tissue-specific, which might depend on chromatin-accessible regions. Moreover, AhR may regulate its own locus chromatin accessibility [164]. On the other hand, AhR may have an indirect impact by other chromatin regulators. Interaction of AhR with the Brahma/SWI2- related gene 1 (BRG1), a subunit of the SWI/SNF chromatin-remodeling complex, affects local chromatin architecture. Depletion of BRG1 reduces of chromatin accessibility. This result has been shown in the regulation of CYP1A1 expression in mouse hepatoma cells, where BRG1 directly targeted to enhancer region [159]. Another study on human retinal pigment epithelial cells revealed similar observation. Moreover, AhR directly interacts with BRG1 [165] and cannot associate with the enhancer in ARNT-deficient cells [159]. DiNatale et al. [166] showed that the expression of IL6 depends on AhR and Brg1 activity. The authors suggest using of an AhR antagonist to inhibit pro-growth and antiapoptotic signaling and sensitize cells to more aggressive treatment heterogeneous mixture of tumors like in the case of carcinoma of the head and neck [166]. Although the abovementioned experiment was conducted on cancer cell lines, in respect of autoimmunity, these outcomes might be interesting because of the treatment with IL-6 inhibitors of various rheumatic diseases, such as rheumatoid arthritis (RA), juvenile idiopathic arthritis (JIA) [167], lupus, Crohn’s disease [168] and necessity of alternative strategies when resistance to biologics drug develop. Interestingly, study conducted on a mouse model of induced-colitis revealed that FICZ prevents intestinal barrier function via AhR activation by suppressing IL-6 and claudin-2 expression [142]. 

Another transcriptional factor that has an impact on chromatin architecture is growth factor independence (GFI1). It has been shown that GFI1 mediates transcriptional repression mainly by recruiting histone-modifying enzymes such as histone deacetylases (HDAC-1–3), histone methyltransferases (EHMT2) or histone demethylases (KDM1A/LSD1/) [169]. GFI1 promoted the development of gut innates lymphocyte (ILC2). Study on ILC2 revealed that AhR suppresses GFI1 recruitment. As a consequence, expression of the interleukin-33 (IL-33) receptor ST2 in ILC2s and expression of ILC2 effector molecules IL-5, IL-13 is downregulated. The authors suggest that AhR is not a general chromatin remodeler but seems to be crucial in the regulation of some event in the genome. Although high expression of AhR in Treg cells in gut (higher than Treg cells in the lymphoid organs and other tissue-resident Treg cells in the lung, fat, skin, and liver [170]) remodeling peak dependent on Ahr was absent in gut Treg cells but present in ILC2s. In other words, gut-ILC2-specific open-chromatin events at the Ahr locus were mostly absent in gut Treg cells. These data support a cell-type-specific positive-feedback role of Ahr in its own transcription regulation of expression distinct cytokines [164]. GFI1 is transiently induced during T cell activation. Furthermore, Zhu et al. [171] showed that GFI-1 is important for the epigenetic regulation of Th17 and in the iTreg-related genes in Th2 cells and activation of GFI-1 inhibits both Th17 and iTreg cell differentiation. Moreover, TGF-β stimulation downregulates GFI-1 [171]. Moreover, it has been proved that Gfi1-deficient mice develop autoimmunity and particularly B cell-dependent autoimmunity like lupus [172]. Gfi1 may also inhibits development of Th17 and Treg. Whereas absence of Gfi-1 is associated with significantly higher percentage of Foxp3+ cells [171]. In the case of lupus, in mice model knockdown of Gfi1 developed this entity which might be also associated with the negative regulation of TLR7. Lack of activation of TLR7 or TLR4 results in decrease of IL-6, IFNβ, TNF production and less productive NF-κB phosphorylation [173]. 

## 5. microRNAs

MicroRNAa (miRNA) are short/small, non-coding RNAs of 19–25 nucleotides long that regulate expression of many genes by the inhibition of translation after complementary base pairing or mRNA degradation after imperfect complementary base pairing with 3’ untranslated regions (3’UTR) of target mRNA [174]. 

According to the primary public repository and online source for microRNA (http://mirbase.org/), human genome encodes over 2600 miRNAs (miRbase v.22) that are involved in post-transcriptional regulation of thousands of protein-coding genes. One miRNA may regulate the expression of several genes and a group of miRNAs may interact with the expression of one gene. Since miRNA controls the expression of variate of genes, they are involved in important life processes including immune cell differentiation, maturation and function [175]. Dysregulations of miRNA expression could lead to autoimmune disease including rheumatoid arthritis, systemic lupus erythematosus, intestinal bowel disease, Sjögren’s syndrome [176,177,178]. Moreover, Hou et al. [179,180] indicated that exposure to environmental chemicals, including AhR agonist TCDD, may alter miRNAs and gene expression that results in diseases and health problems [179,180]. Al-Ghezi et al. [181] demonstrated that mice treated with PTX (pertussis toxin, a microbial metabolite) were characterized by raised serum level of pro-inflammatory cytokines—IL-17A, IL-6 and TNFγ. Moreover, an increased proportion of CD4+ Th1 and Th17 cells in spleen was noted. Contrary to this, mice treated with PTX and additionally with TCDD revealed decreased serum level of above mentioned pro-inflammatory cytokines and increased level of anti-inflammatory cytokines IL-10 and TGF-β. Furthermore, the ratio of Th1 and Th17 cell decreased while Th2 and Tregs increased. Moreover, in CD4+ T cells from mouse spleens treated with PTX and TCDD high expression of miR-3082-5p that targeted IL-17 was observed and low expression of miR-1224-5p that regulate FoxP3. These results suggested that TCDD through AhR activation and miRNA regulation repress inflammation mediated by PTX [181].

According to the available miRNA databases (MIRTarBASE, miRDB- target score >80, Targetscan- miRNA described as “conserved”), almost a hundred miRNAs are potentially involved in post-transcriptional regulation of AhR. However to date, in the aspect of autoimmune diseases, there is still a paucity data regarding the regulation of AhR by miRNA. 

AhR regulates the differentiation of Th17 and Treg cells; therefore, the difference in its expression shifts the balance between these two T cell subtypes and may contribute to the pathogenesis, among others, of Crohn’s diseases (CD). Zhao et al. [182] showed a negative correlation between miRNA and AhR protein level in patients with Crohn’s diseases and that miR-124 may interact with AhR. The expression of miR-124 was high in inflamed colonic and ileac epithelial cells whereas AhR protein level was very low. It was also found that transfection of Caco-2 cells with pre-miR-124 resulted in overexpression of miR-124 and downregulation of AhR. Further analysis confirmed post-transcriptional regulation of AhR by miR-124. Moreover, Caco-2 cells pre-treatment with siRNA for AhR/siAhR and pre-miR-124 stimulated with lipopolysaccharide showed a significantly higher level of few pro-inflammatory cytokines—TNF-α, IL-1β and IL-6. These results suggest that miR-124 promotes the pathogenesis of CD via decreasing AhR [182] and its anti-inflammatory properties have been proved in a clinical trial (ABX464) [183]. Th17 and Treg differentiation may be dependent on AhR ligand and micro-RNA profile. In material isolated from inguinal lymph nodes, Singh et al. [184] observed that dietary AhR ligands such as I3C and DIM promote Treg differentiation and high IL-10 production whereas Th17 promotion was observed after FICZ (endogenous AhR ligand). The observation was interesting because particular signature of microRNA was correlated with the type of T cell. Downregulation of target FOXP3 microRNAs (miR-31, miR-219 and miR-490) and upregulation of miR-495 and miR-1192 (targets of IL-17) was observed in mouse treated with dietary ligands whereas in animals treated with FICZ the opposite expression profile was observed [184].

Recent study conducted by Lu et al. [185] showed that secretion of IL-22, a tissue injured repair cytokine, depends on miR15a/16-1 cluster. It was revealed in a mouse model with immune-mediated liver injury by concanavalin A (ConA) that miR15a/16-1 decreased IL-22 production. In vitro and in vivo study demonstrated that knockout of miR15a/16-1 caused up-regulation of IL-22 in mice CD4+ T cells and ameliorate liver injury via stimulation of cell proliferation and reduced cell apoptosis. It was also noted that AhR, that is essential for IL-22 secretion, is a direct target of miR15a/16-1 in CD4+ T cells. Overexpression of miR15a/16-1 resulted in depletion of IL-22 in CD4+ T cells by inhibiting AhR [185]. 

There is a lot of evidence that AhR plays an important role in rheumatoid arthritis (RA) pathogenesis. In human synoviocytes, AhR ligands upregulate IL-1β whereas in macrophages upregulation of AhR inhibits expression of cytokines TNF-α, IL-1β and IL-6 [186]. Recently *in silico* analysis of the interaction between miRNAs and their potential targets become a popular tool in molecular biology studies. Based on this approach Ogando et al. [187] indicated that translocator of AhR - ARNT is a target of miR-233. Further analysis revealed that in RA derived cells (CD14+) miR-223 is up-regulated. However, pre-treatment THP-1 and HEK-293T cells with pre-miR-223 did not alter AhR protein level but ARNT was reduced. Furthermore, synovial tissue from RA patients was characterized by downregulated level of ARNT and AhR-mediated genes which was the result of miR-223 expression. These results imply complex miR-223-ARNT in human diseases and suggest that proinflammatory cytokines mediated by AhR/ARNT are inhibited by miR-223 [187]. On the other hand, another study on miR-233 showed that its low expression ameliorated the disease severity in the mouse model of collagen-induced arthritis [188]. Therefore, it should be noted that the role of miR-233 in RA is not unambiguous. 

Another example of possible therapy with miRNA activity was miR-155 in systemic sclerosis treatment. It appeared that the expression of miR-155 in skin of SSc patients is upregulated, therefore, Yan et al. [189] suggested its silencing as a promising therapy [187]. Lupus-like phenotypes mice with silenced miR155 were characterized by lower serum levels of IL-4 and IL-17A and authors of this study concluded that miR-155 deficiency reduces the severity of disease course [190]. A few months ago, clinical trials of miR-155 as a potential MS biomarker were registered. miR-155 appears to regulate AhR in signaling pathway linked to B-cell differentiation [191] and also is required for optimum cytokine production by DC to promote Th17 cells, again this ties into a possible AhR involvement. Moreover, miR-155 might be upregulated by multiple proinflammatory cytokines (IL1β, TGFβ, TNFα, IFNγ), which are all targets of AhR-signaling [192]. Comparative study on the microRNA expression profile in serum of patients with different autoimmune connective tissue diseases showed upregulated levels miR-155 and miR-132 in sera of SSc, RA and SLE but not MCTD (mixed connective tissue disease) patients [50]. Deficiency of the miR-132/212 cluster prevented the enhancement of Th17 differentiation by AhR activation [193]. Moreover, in response to the specificity of the gut’s immune system, AhR and miR-212/132 maintain intestinal balance by negative feedback in differentiation of Tr1 cells (type 1 regulatory T cells, which produce IL-10 in a c-Maf-dependent manner) and regulation of IL-10 production [194]. Whereas studies on cholinergic anti-inflammation, conducted by Alzahrani et al. [195] and Hanieh and Alzahrani [196], revealed that activation of AhR by TCDD or GA (novel AhR ligand) upregulate miR-132 or miR212/132 in colitis-associated colon cancer and autoimmune encephalomyelitis. In both entities, authors indicated that silencing of miR-132 exacerbates inflammation [195,196]. A study on colitis and anti-inflammatory response with AhR has been recently conducted by Lv et al. [112]. The authors revealed that miR-302 is dependent on AhR and activation of AhR by alpinetin influences the differentiation of Treg cells but not Th17 [112]. Regulation of miR-302 by AhR has been earlier confirmed by Hu et al. [197]. miR-21 and miR-148 have also been shown to be regulated by AhR activation [198].

Based on in silico analyzes, Ushakov et al. [199], selected miRNAs which may target AhR [197]. Further analysis of the literature shows an association of selected microRNAs with autoimmune diseases, however, they have not been studied in the context of interactions with AhR in these particular entities (Table 2). Nevertheless, in our opinion, there is a high probability that they may have an impact on AhR in the aspect of the immune response. For example, there is a strong possibility that miR-30c may have an inducing role in Th17 differentiation by targeting negative or positive regulators of Th17 differentiation [200].

## 6. Conclusions

Epigenetic mechanisms of AhR and the role of this transcription factor in epigenetic modifications have not been fully explored. Based on the above literature, we can see that it may be implicated into epigenetic processes which are significant in immunomodulation. Although it seems that inhibitors of DNA methylation have a therapeutic significance, in the case of variable autoimmune entities like in lupus treatment, this strategy might be more complicated and needs further research.

AhR regulates the differentiation of Th17 and Treg cells, therefore, the difference in its expression shifts the balance between these two T cell subtypes and may contribute to their pathogenesis of autoimmune diseases. Increasing data reveals AhR cross-roads with the most significant in immunology pathways and probably many of other inter-reaction is still unknown. Therefore, AhR is considered a potential target for new therapeutic strategies in autoimmune diseases. 

Nevertheless, it has to be considered that the implementation of AhR as a therapeutic target might be very complex and many factors, such as type of ligand and its pharmacokinetics, cell type and cell environment (inflammation, tumor) should be taken into account. Another important aspect of effective treatment using AhR as a therapeutic target is drug interactions through activation of CYP1 or phase II enzymes (UGTs, GSTs), as well as different genetic variants of this transcription factor which can affect drug metabolism by enzymes that are regulated by AhR [79]. 

In general, implications of AhR in therapy may be directed into its inhibition as well as activation.

Studies on cancer cell lines revealed upregulated AhR levels [15]. Higher AhR expression has been observed in stomach, thyroid, colon and pancreatic tumors whereas in breast, lung, prostate and cervical cancer AhR mRNA level was similar to non-tumor tissue. In patients with pancreatic tumor increased expression was correlated with patients survival [237]. The intracellular location of AhR activity (nuclear vs. cytosolic) is also important. It seems that nuclear location is associated with disease development and its negative prognosis (kidney tumor [238], urothelial cancer [239]). AhR contributes to carcinogenesis due to CYP1 expression [109]. Moreover, the induction of Treg cells development and differentiation may play a role in immune tolerance in cancer [15]. However, it has been revealed that AhR has both pro-oncogenic and suppressor-like functions depending on the type of tumor [240]. Therefore in the aspect of cancer therapy, different strategies are tried, nevertheless, selective AhR modulators would be preferable in the individualized therapy. Currently conducted clinical trials concern AhR inhibition in tumors, for example, BAY2416964 [241], IK-175 (formerly KYN-175) [242]. Blockage of AhR prevents the activation of immune-tolerant dendritic cells and Treg in tumor microenvironment. On the other hand upregulation of AhR, for example, in the esophageal squamous cell carcinoma cell lines [243], prostate cancer [244] or pancreatic cancer [245] reveals suppressive effect toward cancer. Activation of AhR may be also associated with overcoming of the cell resistance such as methotrexate resistance in acute lymphoblastic leukemia cell [108]. 

Recent identification of structurally-diverse AhR ligands that include salutary phytochemicals and microbial metabolites has changed the course of underlying clinical trials. Nowadays, there is a trend in research on the importance of gut microbiota, their metabolites (including AhR ligands) and various diseases such as MS, nonalcoholic fatty liver disease. The role of diet particularly dietary tryptophan on AhR activation is also comprehensively studied. In the aspect of autoimmune diseases, AhR upregulation and modulation toward increased Treg cell ratio seems to ameliorate of inflammation and inhibit autoimmune response. Particularly in barrier organs regulation of AhR activity reveals high potential of effective therapy. 

AhR ligands, such as coal tar, FICZ and tapinarof via the AhR pathway, restore barrier dysfunction and have been implicated into atopic dermatitis or psoriasis therapy [246,247,248,249,250]. FICZ and UV therapy are recommended as a potential therapeutic agent for scleroderma [251]. Therefore, one of the concepts assumes that the activation of AhR by endogenous ligands may support skin barrier formations, but constitutive, abnormal expression of AhR causes inflammatory skin lesions [252]. Recently, it has been proved that unimpaired AhR signaling is crucial for establishment and maintenance in the neonatal epidermis but also the inflammatory profile of dendritic epithelial T cell in healthy skin [253]. Another well-known example of AhR agonist with a clinical implication is a disease modifying anti-rheumatic drug–leflunomide [254]. Treatment of inflammatory bowel diseases with the AhR agonist, diet and balanced gut microbiota promotes Treg differentiation and attenuates inflammation [91,112,255]. 

Moreover, in the present article, the association between AhR pathway and autoantibodies against complexes such as ACPA or anti-CarP has been described. These autoantibodies are involved in epigenetic modulation and are used in contemporary diagnostic for autoimmune diseases. Noteworthy is that today, in the diagnostics of autoimmune diseases there is a need for more specific and sensitive biomarkers. 

## Figures and Tables

**Table 1:** Characteristic of selected autoimmune diseases and their association with epigenetics mechanisms and aryl hydrocarbon receptor (AhR) presence.

Disease/Abbreviation	Description of Disease [24]	Association with AhR	Other Epigenetic Impacts
Systemic lupus erythematosus (SLE)	Antibodies: ANA, dsDNA and Sm, LA, CL (IgM and IgG), and β2GPI (IgM and IgG); A characteristic skin rash—the so-called butterfly or malar rash—may appear across the nose and cheeks; SLE may affect lupus and can attack any body organ or system, such as joints, kidneys, brain, heart, and lungs.	↑ AhR in PBMCs and Th17 cells, AhR ratio Th17/Treg positively correlated with disease activity [25]	Hypomethylation in CD4+ T-cell [26] Hypomethylated neutrophils and granulocytes [27] ↓ DNMT1 and ↓ MBD4 in CD4+[26] ↑ STAT3 through histone acetylation (p300) [28] ↓ HDAC6 in comparison to HC [29]
Rheumatoid arthritis (RA)	Autoantibodies: RF, ACPA An inflammatory disease that causes pain, swelling, stiffness, and loss of function in the joints	↑ AhR in synovial tissue (higher than in osteoarthritis) [30].	Synovial fibroblast: ↑H3K4me3 are increased and ↓ H3K27me3 in the MMP promoters [31] ↑ IL-6 as a result of H3ac [32] HATs/HDACs disbalance [33] Global hypomethylation in PBMC [34]
Systemic sclerosis (SSc)	Centromere (CENP-B), topoisomerase I (Scl-70), and RNApol III SSc includes the skin but also involves the tissues beneath (blood vessels and major organs). There are two types SSs: limited (lSSc) and diffuse (dSSc). Within skin layers collagen excess, calcium deposits (calcinosis); patients reveal Raynaud’s phenomenon, esophageal dysfunction	cross-talk AhR-TGFβ and fibrosis [35] ↑ AhR mRNA in PBMC; ↑ AhR mRNA in d SSc than that of lSSc [36]	Global DNA hypomethylation was observed in the CD4+ T cells [26,37] ↑ methylation the Fli1 promoter region may induce excessive collagen transcription in SSc fibroblasts [38] in serum of SSc ↓ anti-HDAC-3 antibodies [39] ↑ HDAC-1 and ↑ HDAC-6 in dermal fibroblasts and suppression of the acetylated forms of histones H3 and H4 in the Fli1 promoter of SSc fibroblasts [40]
Psoriasis (PSO)	a skin disease characterized by scaling and inflammation; About 15% of people with psoriasis have joint inflammation that produces arthritis symptoms (psoriatic arthritis)	↓ AhR exacerbate psoriasis [41]	Global hypomethylation in affected skin [42] ↑ HDAC1 in the skin [43] ↓ H3 and ↓ H4 acetylation and increased ↑ H3K4 methylation in PBMC [44]
Sjögren Syndrome (SS)	SSA/Ro autoantibodies attack and destroy the moisture-producing glands (tear, salivary) but sometimes extraglandular involvement occurs	AhR potentially induce the reactivation of EBV in SS patients in both B cells and salivary epithelial cells [45,46]	↓ DNMT in epithelial cells from salivary gland [47] Hypermethylation of FOXP3 in CD4+ cells [48] Hypomethylation of MX1, IFI44L, PARP, IFITM1, TNF and their ↑ expression in B cells [47]
Mixed connective tissue disease (MCTD)	U1-RNP describe overlapping groups of connective tissue disorders; characterized by joint pain; muscle weakness; cardiac, lung and skin manifestations; kidney disease; and dysfunction of the esophagus	?	DNAm ↓ in regions transcriptionally responsive to the presence of interferon or involved in type I interferon pathways [49] ↓miR-126 [50]
Crohn’s Disease (CD)	ASCA symptoms are similar to other intestinal disorders such as irritable bowel syndrome and to another type of IBD called ulcerative colitis; Most often inflammation occurs in the small intestine, marked by patchy areas of full-thickness inflammation	TCDD-activated AhR associated with demethylation of FOXP3 and hypermethylation IL-17 promoters [51] G-allele of rs7796976 in AhR increased the risk for disturbed intestinal permeability [52]	↑ methylation in α-defensin (antimicrobial peptides, potential CD biomarker) and ↓ methylation in TNFα [53]
Ulcerative colitis (UC)	ANCA A nonspecific inflammatory disease of the bowel in the top layers of the lining of the large intestine rarely affects the small intestine except for the lower section	activation IL-22 pathway through Ahr reveals therapeutic effect continuous activation of the IL-22 pathway increases the risk of colitis-associated cancer [54]	IL-17A (IVS1+18) and STAT4 (rs7574865) the T risk allele-loss of CpG sites-↑ expression of IL-17A and STAT4 in PBMCs and colonic tissue [55,56] Hypermethylation of TRAF6 in PBMC [57] ↑ IFN-γ gene methylation in PB T cells [58] global hypomethylation in rectal mucosa [59] ↑ histone H3 acetylation within Foxp3 loci in Treg cells [60]
Type 1 diabetes (T1D)	Insulin-dependent diabetes mellitus (IDDM) (T1D) most often develops in children and young adults	treatment with TCDD suppressed spontaneous and progressive destruction of β cells in NOD mouse model of T1D and prevented diabetes in NOD model via AhR activation [61]	Hypermethylation of FOXP3 in CD4+[62] ↑ H3K9me2 in CTLA4 (T1D susceptibility gene) correlates with T cell activation [63]
Grave’s disease	A glandular autoimmune disease affecting the thyroid gland with an increase in the production of thyroid hormone	↑ AhR, ↑ Th22 cells, ↑ serum IL22 level in comparison to HC [64]	Hypermethylation of TSHR in thyroid cells [65] ↑ HDAC1, ↑HDAC2 and ↓H4ac in PBMC [66] Hypermethylation of CAMI, CD247, CTLA4 associated with T cell signaling [67]

ANA, antinuclear antibodies; ANCA, antineutrophil cytoplasmic antibodies; ACPA, anticitrullinated protein. antibodies; β2GPI, β2 glycoprotein 1; CL, cardiolipin; LA, lupus anticoagulants; RF, rheumatoid factor; RNP, ribonucleoprotein; ↑, upregulation (increased); ↓, downregulation (decreased); ?, unknown association.

**Table 2:** microRNA associated with AhR and their impact on selected autoimmune diseases.

	Connection with AhR [Ref]	Autoimmune Disease	microRNA Association with Autoimmune Disease [Ref]
miR-28	[199]	pSS	[201]
miR-30c	[199]	MS	[202]
		pSS	[201,203]
		IBD	[204]
miR-30e	[199]	MG	[205,206]
miR-139	[199]	RA	[207]
miR-153	[199]	LE	[208]
mir-181a	[209]	SLE	[50,210]
		RA	[50]
		pSS	[211]
		CD	[212]
mir-29a	[213] [214]	SLE	[50,215]
		RA	[50,216]
		UC	[217]
		pSS	[218]
mir-29b	[219]	SLE	[221]
	[220]	MS	[222]
mir-378a	[213]	UC	[223]
		pSS	[201]
mir-203	[224]	RA	[225]
		SLE	[210]
		PSO	[226]
		UC	[227]
mir-196a	[228]	SSc	[229,230]
mir-490	[184]	RA	[231]
mir-148a	[112]	SLE	[232]
		RA	[233]
mir-31	[184]	SLE	[234]
		PSO	[235]
		pSS	[236]

pSS-primary Sjögren Syndrome, MS-multiple sclerosis, MG-myasthenia gravis, CD-Crohn’s disease; PSO-Psoriasis; UC-ulcerative colitis; SSc-systemic sclerosis.
